# Effect of Nanoalumina on Sex Hormones and Fetuses in Pregnant Rats

**DOI:** 10.5935/1518-0557.20210045

**Published:** 2022

**Authors:** Hossein Soltaninejad, Hadi Zare-Zardini, Mohammad Amir Amirkhani, Masoomeh Mohammadzadeh, Akram Ghadiri-Anari, Mahtab Ordouei, Ashraf Alemi, Adel Ghorani-Azam

**Affiliations:** 1 Department of Nanobiotechnology, Faculty of Biological Sciences, Tarbiat Modares University, Tehran, Iran; 2 Stem Cell and Regenerative Medicine Institute, Tehran University of Medical Sciences, Tehran, Iran; 3 Hematology and Oncology Research Center, Shahid Sadoughi University of Medical Sciences, Yazd, Iran; 4 Department of Sciences, Farhangian University, Isfahan, Iran; 5 Medical Nanotechnology and Tissue Engineering Research Center, Yazd Reproductive Sciences Institute, Shahid Sadoughi University of Medical Sciences, Yazd, Iran; 6 Research and Clinical Center for Infertility, Shahid Sadoughi University of Medical Sciences, Yazd, Iran; 7 Department of Internal Medicine, Diabetes Research Center, Shahid Sadoughi University of Medical Sciences, Yazd, Iran; 8 Department of Pediatrics, Shahid Sadoughi University of Medical Sciences, Yazd, Iran; 9 Department of Biochemistry, Abadan Faculty of Medical Sciences, Abadan, Iran; 10 Department of Forensic Medicine and Toxicology, School of Medicine, Urmia University of Medical Sciences, Urmia, Iran

**Keywords:** nanoalumina, fetuses, sex hormones, size, toxicity

## Abstract

**Objective:**

This study aimed at investigating the effect of nanoalumina on sex hormones, and fetuses in pregnant rats.

**Methods:**

In this study, sixty-four pregnant rats were divided into eight groups. The control and the injection-control group received normal food and water, and 0.5 ml of distilled water, respectively. Treatment groups were treated with 25, 50, 100, 250, 500, and 1000µg/ml concentrations of Nanoalumina from the 7^th^ day until the 18th day of pregnancy. On the 18^th^ day, the rats were investigated in terms of their hormone levels. We evaluated the number of healthy and aborted offspring, as well as fetus size.

**Results:**

Nanoalumina caused an increase in progesterone hormones at the concentrations of 250, and 500µg/ml, and a significant reduction in estrogen hormone and aborted fetuses at the concentrations of 250 and 500µg/ml (*p*<0.05). The largest and smallest size of fetuses were observed in 500µg/ml and 1000µg/ml, respectively. The highest number of aborted fetuses was observed in the group treated with the 500µg/ml concentration. There was no aborted fetuses with 25, 50,100, control, and injection-control groups.

**Conclusions:**

Due to nanoalumina toxicity, it must be used with caution.

## INTRODUCTION

Recently, nanostructures have found increased applications in technology, research and medicine (Liang *et al*., 2012; Bellah *et al*., 2012). These compounds have unique properties because of their small size. In the past decades, nanotechnology has been increasingly used in sciences due to their wide range of biomedical applications, for example in coating, drug delivery, medical imaging, and etc. (Latysh *et al*., 2006; Boncel *et al*., 2012; He *et al*., 2010; Amiri *et al*., 2012; Zardini *et al*., 2012; 2014). In addition to its beneficial properties, nanostructures have dangerous toxicity, especially on humans (Zare-Zardini *et al*., 2015a; b). For some types of particles, the smaller they are, the greater their surface area to volume ratio and the higher their chemical reactivity and biological activity (Leslie-Pelecky & Rieke, 1996; Hahn, 2003). The toxicity of different nanostructures has been proven on different organs, such as blood, lungs, liver, skin, gut, and etc (Faqi *et al*., 2008). Once in the blood stream, nanostructures can be transported around the body and be taken up by organs and tissues, including the brain, heart, liver, kidneys, spleen, bone marrow and nervous system (Tseng *et al*., 2008).

Nanomaterials are toxic for human tissues and in cell-cultures, resulting in increased oxidative stress, inflammatory cytokine production and cell death. Nanostructures may be taken up by cell mitochondria in the cell nucleus. Nanomaterials can cause DNA mutation and induce major structural damage to the mitochondria. One organ affected by nanostructures is the sexual organ. Here, it is important that nanostructures can penetrate deeper into skin layers and possibly be absorbed into the systemic circulation and build up in tissues, especially in sexual organs. Alumina is one of the inert biomaterials used in biomedicine (El-S'adany *et al*., 2013; Affatato *et al*., 2012). This nanostructure has been defined as a suitable compound to be used in different fields of life.

The aim of the present study was to prepare nanoalumina by the sol-gel method and investigate its toxicity on changes of sex hormone and abortion in pregnant rats.

## MATERIALS AND METHODS

According to our previous study (Mehregan *et al*., 2015), we used the sol-gel method to synthesize the nanostructures; and we used X-Ray Diffraction (XRD) and transmission electron microscope (TEM) to identify the crystalline mineralogical phases of the powders and the micrographs, respectively. We randomly divided eighty female rats into 8 groups, including six treatment groups, one control group, and one injection control group. Since the formation of a vaginal plug (G0), pregnant female rats were maintained *in vitro* for 7 days. Different concentrations of nanostructures (25, 50, 100, 250, 500, and 1000µg/ml) were intraperitoneally injected every day from the seventh day of pregnancy until the eighteenth day. Table 1 depicts the summarized information from the 8 groups.

**Table 1.** Details from the 8 groups in this study that were treated with different concentrations of nanoalumina.

Groups	
Control	Normal condition, water, regular food
Injection Control	Injection of distilled water
Treatment group 1	Injection of 0.5ml nanopowder (25µg/ml)
Treatment group 2	Injection of 0.5ml nanopowder (50µg/ml)
Treatment group 3	Injection of 0.5ml nanopowder (100µg/ml)
Treatment group 4	Injection of 0.5ml nanopowder (250µg/ml)
Treatment group 5	Injection of 0.5ml nanopowder (500µg/ml)
Treatment group 6	Injection of 0.5ml nanopowder (1000µg/ml)

On the 18^th^ day of pregnancy, we assessed the rats' hormone levels, the number of healthy embryos, and aborted fetuses. After the injection, we took blood samples from the rats' hearts every other day up to the 18^th^ day of pregnancy. Using biochemical tests, we compared the rats considering their levels of estrogen and progesterone hormones. Other variables investigated include the number of healthy embryos and the number of aborted fetuses.

## RESULTS

**Figure 1:**
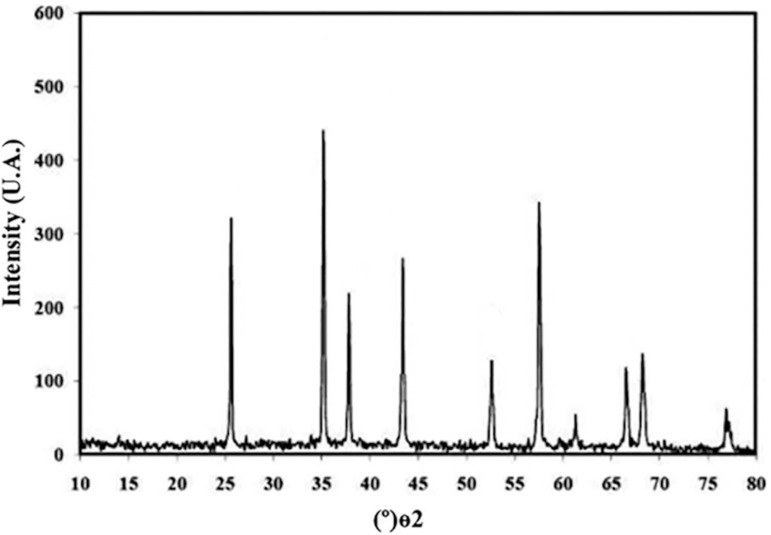
The XRD image nanoalumina.

**Figure 2:**
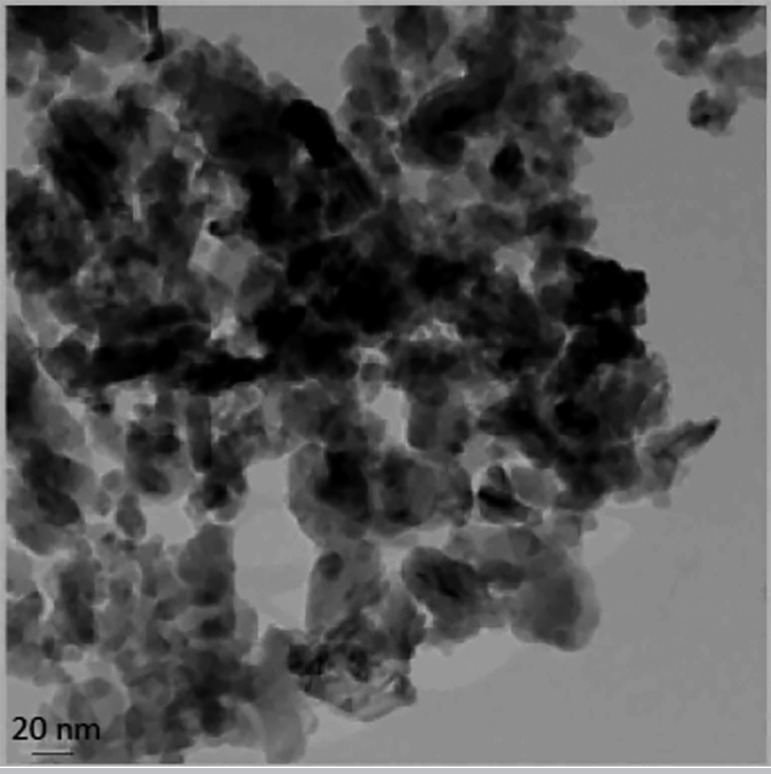
TEM image of nanoalumina.

We used the sol-gel method to synthesize alumina nanoparticles. The resulting nanoalumina powders were characterized by X-ray diffraction and scanning electron microscopy (TEM). Figure 1 shows the XRD pattern of alumina powder. The peak broadening at lower angle in Figure 1 is more meaningful for the calculation of particle size; therefore we calculated the size of the nanocrystals with the Debye-Scherrer formula, using reflection from the XRD pattern. Figure 2 shows the transmission electron microscopy (TEM) micrographs from the alumina powder obtained from the aluminum chloride solution (0.1M), heat-treated at 100ºC for 24 hours. There are two types of particles with different geometries, namely: needle-shaped particles with average particle size below 30nm and spherical particles, with average size below 20nm.

**Figure 3:**
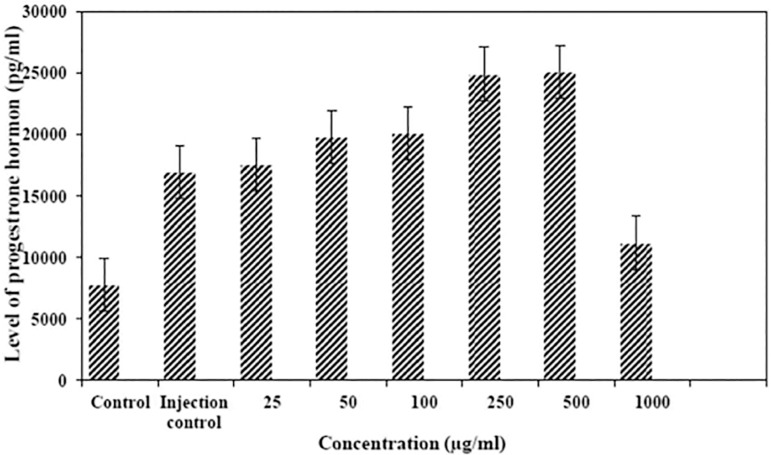
Comparison of progesterone levels among the eight groups.

**Figure 4:**
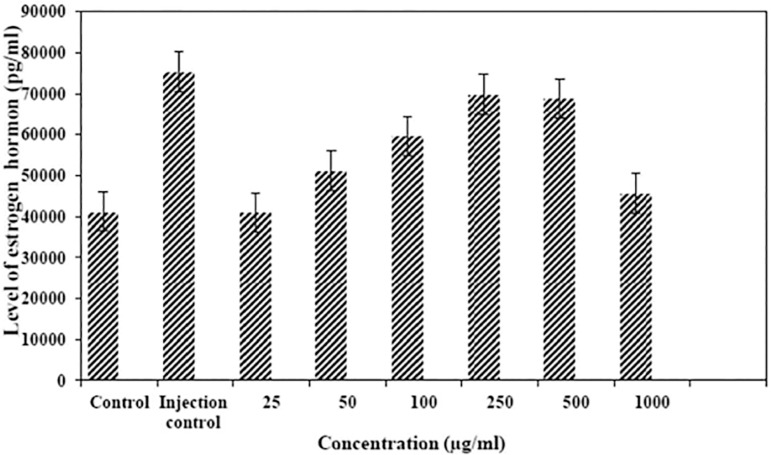
Comparison of estrogen levels among the eight groups.

Among six treatment groups, the highest level of progesterone hormone was in the group treated with the Al_2_O_3_ nanostructure at a concentration higher than 500µg/ml (25150±3855pg/ml/24). The lowest level of progesterone hormone was seen in the control group in comparison with six treatment groups and the injection control group (7800±812pg/ml) (Figure 3). According to our results, there were significant differences among the groups (*p*<0.05). The hormone raise was consistent with the increase in nanostructure concentration, up to 500µg/ml (*p*<0.05). At 1000µg/ml, the level of progesterone hormone was decreased. Estrogen among the treated groups was highest in the group treated with 250 and 500µg/ml (69850 and 68750pg/ml in 250 and 500µg/ml, respectively). The injection control group had the highest estrogen level (75400±3723pg/ml) (Figure 4). There was no significant difference involving the group treated with the concentration of 250 and 500 µg/ml. The difference between the control group and the injection-control group, 250 and 500 ppm groups was significant, regarding reduction in estrogen hormone level (*p*<0.001). The difference between the 1000µg/ml group and the injection control group, the 250 and 500µg/ml groups, and the control group was significant regarding reduction in estrogen hormone level (*p*<0.05).

In higher concentrations of nanostructures (1000µg/ml), the reduction of two hormones was lower than in other treated groups. The results showed that the number of healthy embryos was highest at 25, 50 and 1000µg/ml concentrations in comparison with other concentrations, e.g. 100, 250 and 500µg/ml. The number of healthy embryos was 15 and 14 in control group and in the injection control group, respectively (Table 2). There were no significant differences among the groups regarding the number of healthy embryos.

**Table 2.** Results from the number of healthy and aborted fetuses in eight groups.

Groups	Healthy Fetus	Aborted Fetus
Control	15	0
Injection Control	14	0
Nanoalumina (25µg/ml)	15	0
Nanoalumina (50µg/ml)	14	0
Nanoalumina (100µg/ml)	15	0
Nanoalumina (250µg/ml)	10	2
Nanoalumina (500µg/ml)	8	12
Nanoalumina (1000µg/ml)	13	3

As shown in this table, all the groups were different regarding the number of aborted fetuses. The highest number of aborted fetuses was in the group treated at 500µg/ml concentration (12 aborted fetuses). There were no aborted fetuses in the 25, 50, 100, and in the control groups. There were no aborted fetuses in the injection control group. There was a significant difference among the groups regarding the mean number of aborted fetuses. The difference between the control and the injection control group as well as the 100, 250 and 500µg/ml and the injection control group was significant regarding the mean value of aborted fetuses (*p*<0.05). The difference between the 1000µg/ml and the injection control group, 150, 250 and 500µg/ml groups was significant regarding the mean number of aborted fetuses (*p*<0.05).

**Figure 5:**
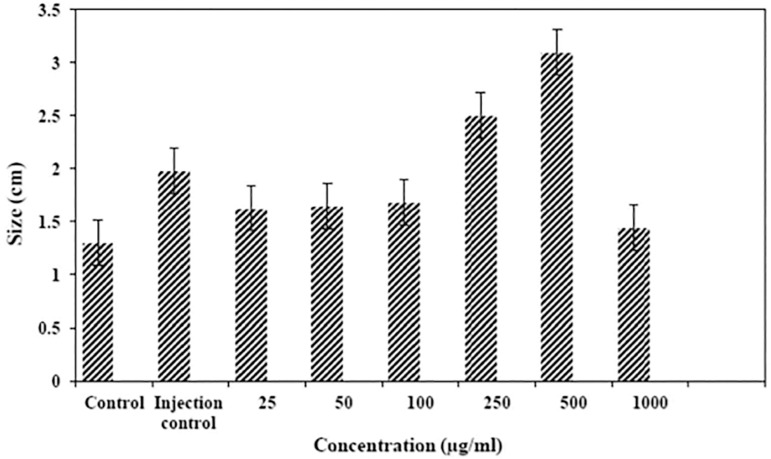
Comparison of average total size of fetuses among the eight groups.

According to acquired data, the largest and smallest sizes of fetuses were seen in the 500µg/ml and the 1000µg/ml of nanostructures, respectively. Between all groups, the smallest size belonged to the control group. The reduction in fetus size was significant between the control and the injection control groups, 100, 250 and 500µg/ml groups (*p*<0.05). The increase of size was significant between the 500 and 1000µg/ml groups (*p*<0.05) (Figure 5).

## DISCUSSION

Nanomaterials have toxicological properties in comparison to the same substance in the bulk form (Zhang *et al*., 2014). The use and release of nanostructures into the environment can have effects on each stage of the life cycle (Palombo *et al*., 2014). The toxicity is a function of particle number and surface area, rather than mass. The knowledge about the toxicity of nanomaterials is incomprehensible, especially long-term environmental impacts and chronic health impacts (Sharifi *et al*., 2012). These nanomaterials can cause defects on all organs of the body such as liver, blood, brain, lung and etc. (Kermanizadeh *et al*., 2015; Kumar *et al*., 2014; Zhao & Castranova, 2011; Soleymani *et al*., 2014). In this study, we investigated the effects of nanoalumina on changes in estrogen and progesterone levels, the total size of the fetus and abortion. We assessed the effects of different concentrations of nanoalumina on progesterone, and it showed changes in hormone levels compared to the control group in a way that treatment with concentration of 500µg/ml led to the highest amount of change in progesterone and other concentrations, which decreased the levels of progesterone. Considering both treatment and control groups, the lowest level of progesterone was in the control group.

The increase found in the group treated with concentration of 250 and 500µg/ml was statistically significant (*p*<0.05) in comparison with all other groups. According to the acquired data, we can conclude that these examined concentrations have the ability to cause changes in hormone levels and these changes were more obvious among the 250 and 500µg/ml concentrations. This suggests that increases in concentrations can affect hormone levels only up to a certain extent. Considering changes in the estrogen level after being treated with six concentrations of nanoalumina, we noticed that nanoalumina change estrogen hormone levels in a way that lower concentrations (25, 50, 150, 300 and 500µg/ml) increase estrogen and higher concentrations (1000µg/ml) decreased estrogen levels in comparison with the control group. The decline in the level of estrogen in the control groups was significant compared to the three other groups treated with 100, 250 and 500µg/ml concentrations of nanoalumina (*p*<0.002).

The lowest estrogen level was in the group treated with nanoalumina at 1000µg/ml concentration in comparison with five other treatment groups, and this different was statistically significant (*p*<0.05). Therefore, we may suppose that the increase in nanoalumina concentration from 500µg/ml to 1000µg/ml can reduce estrogen level. According to the results obtained, we could say that nanoalumina may increase estrogen levels only up to certain concentrations when compared to the control group, and in higher concentrations it reduces the estrogen level. Given that recent studies proved that silver nanoparticles can have different effects on various body parts of living organisms, we can suppose that the level of these nanostructures bring about changes to estrogen levels; however, the exact mechanism has not been examined yet. Regarding the number of healthy embryos after treatment with nanoalumina, there were changes in each group.

Nanoalumina reduced the number of healthy embryos in a way that the number of healthy embryos was 8 at the concentration 500µg/ml. The difference between 500 and 1000µg/ml was not significant (*p*>0.05) (Figure 3). Overall, we can conclude that high concentrations of nanoalumina (500µg/ml) have a significant effect on reducing the number of healthy embryos. Considering this data and other reports, the use of nanostructures must bear more caution. Hormonal levels changes in pregnant females can affect menstrual function, fertility, and fetus health (Scsukova *et al*., 2015). Similar studies showed that nanoparticles change the hypothalamic-pituitary-ovarian axis function and cause female infertility. The buildup of these nanoparticles can also cause abortion. Size, exposure time and concentration have significant effects on the severity of side effects. We obtained similar results in our study (Rollerova *et al*., 2015; Oberdörster *et al*., 2004; Liu *et al*., 2016; Kong *et al*., 2016). We also showed a significant and concentration-dependent effect of nanoalumina on changes in sex hormones.

As mentioned above, hormonal changes in the treatment groups were more severe than in the control groups; although these changes were also seen among the injection control group. Therefore, considering the effective changes in the injection control group, the significant effects of nanoalumina injection on sex hormones does not seem to be justified. But the big difference between the nano-treated groups compared to the injection control and control groups is the number of aborted fetuses, which is very significant in the 500 and 1000µg/ml of nanoalumina. Therefore, due to the effective hormonal changes in the injection control group, it may not be possible to justify the hormonal changes caused by the nanoalumina injection; however, the high level of abortion in the nano-treated groups can prove the risk of high doses of this nanoparticle. Yang *et al*. (2018) showed that gold nanoparticles can induce abortion by inhibiting ectodermal differentiation. This group indicted that treatment with gold nanoparticles during early pregnancy causes significant increases in the rate of abortions and stillbirths (Yang *et al*., 2018). Similar results showed that sex hormone imbalances can increase the risk of spontaneous abortion in pregnant rats (Li *et al*., 2013). We showed similar data in our study.

Therefore, according to the results of our study on the significant effect of different concentrations of nanoalumina on sex hormones and in particular - increasing the number of abortions, the use of this nanoparticles, especially in high doses in different products, should be more cautious.
